# Supplementary material for: Reactive nitrogen restructures and weakens microbial controls of soil N2O emissions
Source: Commun Biol. 2022 Mar 28;5:273. doi: 10.1038/s42003-022-03211-4 (PMC8960841; doi:10.1038/s42003-022-03211-4)
Supplement: Supplementary file 2 — Supplementary Information [file 42003_2022_3211_MOESM2_ESM.pdf]

Supplemental Figures and Tables

Supplementary Table 1. Long-term fertilization trials. Site, starting year, number of replicated plots and corresponding nitrogen application rates at 14 sampled long-term field trials located at 11 sites across Sweden

Location	Year of establishment	Replicates	Fertilization (kg ha^{-1})	Coordinates	Reference
Ultuna	1956	4	0-80	59°48'N 17°48'E	Kirchmann, Persson [1]
Röbäcksdalen	1980	4	0-120	63°49'N 20°13'E	Carlgren and Mattsson [2]
Säby 1	1970	4		59°49'N 17°42'E	Poeplau, Kätterer [3]
Lanna 1	1981	4		58°21'N 13°08'E	Poeplau, Kätterer [3]
Lönnstorp	1980	4		55°40'N 13°05'E	Poeplau, Kätterer [3]
Kungsängen	1963	2	0-120	59°29'N 17°40'E	Carlgren and Mattsson [2]
Fors	1963	2		60°20'N 17°29'E	Carlgren and Mattsson [2]
Bjertorp	1966	2		58°14'N 13°08'E	Carlgren and Mattsson [2]
Säby 2	1969	6	0-135	59°49'N 17°42'E	Persson, Bergkvist [4]
Lanna 2	1965	6		58°20'N 13°07'E	Persson, Bergkvist [4]
Stenstugu	1968	6		57°36'N 18°26'E	Persson, Bergkvist [4]
Lanna 3	1996	4	0-80	58°34'N 13°10'E	Kätterer, Börjesson [5]
Borgeby	1957	2	0-150	55°44'N 13°03'E	Williams, Börjesson [6]
Västerås	1998	4	0-100	59°37'N 16°33'E	Svensson, Odlare [7]

Supplementary Table 2. Potential activity measurements and denitrification end-product ratio in fertilized and unfertilized soils across sites (mean values, expressed as ng N_2O-N g^{-1} soil DW min^{-1} with standard deviation). Significant effects of fertilization treatment and site were determined by two-way ANOVA, and significant effects ($p \leq 0.05$) are presented in bold.

Activity	Unfertilized	Fertilized	Fertilization	Site	Fertilization × Site
Total denitrification[a]	15.32 ± 6.50	18.68 ± 7.83	**$F_{1,80}$ = 11.88*****	**$F_{13,80}$ = 11.72*****	$F_{13,80}$ = 1.15
N_2O emissions[a]	6.28 ± 3.15	9.14 ± 4.68	**$F_{1,80}$ = 40.26*****	**$F_{13,80}$ = 19.79*****	$F_{13,80}$ = 1.38
$N_2O/(N_2+N_2O)$	0.43 ± 0.17	0.53 ± 0.24	**$F_{1,80}$ = 11.15****	**$F_{13,80}$ = 12.96*****	$F_{10,86}$ = 1.56

[a]Values Box-Cox transformed prior to ANOVA analysis
$^{***}p < 0.001$
** $0.001 < p < 0.01$
* 0.01 < *p* 0.05

Supplementary Table 3. Soil physico-chemical properties of fertilized and unfertilized soils across all sites. Nutrients have been determined using both ammonium-lactate and HCl extraction method (denoted as AL and HCl, respectively). Significant effects of fertilization treatment, sampling site and interaction effects determined by 2-way ANOVA are shown in bold text.

**Soil factor**	**Unfertilized**	**Fertilized**	**Two-way ANOVA (*F*-ratio$_{df}$)**		
	(Mean ± SD)		Fertilization	Site	Fertilization x Site
pH	6.57 ± 0.46	6.57 ± 0.47	$F_{1,80}$ = 0.00	**$F_{13,80}$ = 63.48*****	**$F_{13,80}$ = 2.42****
P AL [mg/100g][a]	11.61 ± 6.59	9.89 ± 5.43	**$F_{1,80}$ = 8.74****	**$F_{13,80}$ = 32.75*****	$F_{13,80}$ = 0.55
K AL [mg/100g][a]	17.16 ± 5.37	16.21 ± 5.3	**$F_{1,80}$ = 3.96***	**$F_{13,80}$ = 32.21*****	$F_{13,80}$ = 0.53
Mg AL [mg/100g][a]	24.63 ± 14.20	23.2 ± 13.78	$F_{1,80}$ = 2.47	**$F_{13,80}$ = 80.28*****	$F_{13,80}$ = 0.58
Ca AL [mg/100g][a]	287 ± 343	307 ± 373	**$F_{1,80}$ = 5.39***	**$F_{13,80}$ = 71.94*****	$F_{13,80}$ = 0.59
Al AL [mg/100g][a]	31.5 ± 14.45	31.76 ± 15.98	$F_{1,80}$ = 0.06	**$F_{13,80}$ = 57.73*****	$F_{13,80}$ = 1.08
Fe AL [mg/100g][a]	64.46 ± 23.21	59.52 ± 24.79	**$F_{1,80}$ = 6.25***	**$F_{13,80}$ = 40.85*****	$F_{13,80}$ = 0.81
Cu HCl [mg/100g]	16.54 ± 7.12	16.47 ± 7.24	$F_{1,80}$ = 0.06	**$F_{13,80}$ = 202.71*****	$F_{13,80}$ = 1.09
Humus (%)	2.09 ± 0.94	2.44 ± 0.98	**$F_{1,80}$ = 17.98*****	**$F_{13,80}$ = 34.65*****	$F_{13,80}$ = 1.19
Clay (%)[a]	27.71 ± 12.03	27.29 ± 12.07	$F_{1,80}$ = 0.56	**$F_{13,80}$ = 102.85*****	$F_{13,80}$ = 1.09
Silt (%)	44.8 ± 14.1	45.18 ± 14.27	$F_{1,80}$ = 0.51	**$F_{13,80}$ = 199.82*****	**$F_{13,80}$ = 1.91***
Sand (%)[a]	25.43 ± 17.42	25.16 ± 17.82	$F_{1,80}$ = 0.17	**$F_{13,80}$ = 102.40*****	$F_{13,80}$ = 0.72
Dry weight [g/kg]	795 ± 36	790 ± 39	**$F_{1,80}$ = 7.16***	**$F_{13,80}$ = 101.33*****	$F_{13,80}$ = 1.39
C_{org} [g/kg][a]	13.98 ± 4.59	15.44 ± 4.42	**$F_{1,80}$ = 22.11*****	**$F_{13,80}$ = 46.82*****	$F_{13,80}$ = 0.8
C/N[a]	11.72 ± 1.72	11.24 ± 1.91	**$F_{1,79}$ = 7.45****	**$F_{13,79}$= 19.68*****	**$F_{13,79}$ = 2.86****
N_{total} [g/kg][a]	1.43 ± 0.38	1.64 ± 0.34	**$F_{1,79}$ = 52.23*****	**$F_{13,79}$= 24.95*****	**$F_{13,79}$ = 4.66*****
NH_4^+ [mg/kg][a]	2.87 ± 1.22	3.37 ± 1.11	**$F_{1,79}$ = 12.41*****	**$F_{13,79}$= 8.18*****	**$F_{13,79}$ = 2.27*****
NO_3^- [mg/kg]	2.03 ± 1.15	3.18 ± 2.62	**$F_{1,79}$ = 14.52*****	**$F_{13,79}$= 20.64*****	**$F_{13,79}$ = 2.43*****

[a]Values Box-Cox transformed prior to ANOVA analysis
$^{***}p < 0.001$
** $0.001 < p < 0.01$
* $0.01 < p$ 0.05

Supplementary Table 4. Gene abundances in fertilized and unfertilized soils across sampling sites, expressed as mean ± standard deviation × 10^8 copies per gram dry soil. Significance of fertilization, site and interaction effects were determined by two-way ANOVA, and significant *F*-ratios (p < 0.05) are shown in bold.

Gene	Unfertilized	Fertilized	Fertilization	Site	Fertilization × Site
16S rRNA[a]	47.27 ± 13.25	56.17 ± 16.42	**$F_{1,80}$ = 24.20*****	**$F_{13,80}$ = 12.98*****	**$F_{13,80}$ = 2.24***
nirK[a]	0.67 ± 0.27	0.88 ± 0.45	**$F_{1,79}$ = 18.63*****	**$F_{13,79}$ = 10.23*****	**$F_{13,79}$ = 2.32****
nirS[a]	3.96 ± 1.84	4.33 ± 1.93	$F_{1,80}$ = 2.44	**$F_{13,80}$ = 9.85*****	$F_{13,80}$ = 0.62
nosZI	0.38 ± 0.16	0.46 ± 0.19	**$F_{1,80}$ = 12.37*****	**$F_{13,80}$ = 12.96*****	$F_{13,80}$ = 1.55
nosZII	0.08 ± 0.04	0.09 ± 0.04	$F_{1,80}$= 3.50	**$F_{13,80}$ = 10.55*****	$F_{13,86}$ = 1.66
(*nosZI*+*nosZII*)/ (*nirK* + *nirS*)	0.10 ± 0.03	0.11 ± 0.04	$F_{1,79}$= 1.84	**$F_{13,79}$ = 7.79*****	$F_{13,86}$ = 0.72

$^{***}p < 0.001$
** $0.001 < p < 0.01$
* $0.01 < p$ 0.05
[a]Box-Cox transformed prior to analysis

Supplementary Table 5. Diversity of communities of frequent OTUs in fertilized and unfertilized soils across sampling sites (mean ± standard deviation). Significance of fertilization, site and interaction effects were determined by two-way ANOVA, and significant *F*-ratios (p < 0.05) are shown in bold.

	Unfertilized	Fertilized	Fertilization	Site	Fertilization × Site
Shannon's *H′*	6.23 ± 0.14	6.26 ± 0.15	**$F_{1,77}$ = 8.61****	**$F_{13,77}$ = 30.52*****	**$F_{13,77}$ = 4.01*****
Richness	1290.20 ± 93.73	1281.44 ± 128.52	$F_{1,77}$ < 0.01	**$F_{13,77}$ = 12.28*****	**$F_{13,79}$ = 2.04***
Phylogenetic diversity	118.99 ± 6.57	118.40 ± 8.75	$F_{1,77}$ < 0.01	**$F_{13,77}$ = 10.44*****	**$F_{13,79}$ = 2.02***

$^{***}p < 0.001$
** $0.001 < p < 0.01$
* $0.01 < p$ 0.05

Supplementary Table 6. Primers and thermal conditions for qPCR. Quantified genes include bacterial 16S rRNA, the functional genes for denitrification (*nir*) and N_2O reduction (*nosZ*)

Gene	Primer sequence (5'-3')	Amplicon length	Thermal conditions	Reference
16S rRNA	341F CCTACGGGAGGCAGCAG 534R ATTACCGCGGCTGCTGGCA	194 bp	(95°C 5 min)x1 (95°C 15s, 60°C 30s, 72°C 30s, 78°C 10s)x35	Lopez-Gutierrez, Henry [8]
nirS	Cd3aF AACGYSAAGGARACSGG R3cd GASTTCGGRTGSGTCTTSAYGA	426 bp	(95°C 7 min)x1 (95°C 15s, (65-60°C – 1°/cycle) 30s, 72°C 30s) x6 (95°C 15s, 60°C 30s, 72°C 30s, 80°C 15s)x35	Throback, Enwall [9]
nirK	nirK 876F ATYGGCGGVAYGGCGA nirK 1040 GCCTCGATCAGRTTRTGGTT	165 bp	(95°C 7 min)x1 (95°C 15s, (63-58°C – 1°/cycle) 30s, 72°C 30s) x6 (95°C 15s, 58°C 30s, 72°C 30s, 80°C 15s) x35	Henry, Baudoin [10]
nosZI	1840F CGCRACGGCAASAAGGTSMSSGT 2090R CAKRTGCAKSGCRTGGCAGAA	267 bp	(95°C 7 min)x1 (95°C 15s, (65-60°C – 1°/cycle) 30s, 72°C 30s) x6 (95°C 15s, 60°C 30s, 72°C 30s, 80°C 15s)x35	Henry, Bru [11]
nosZII	nosZ-II_F CTIGGICCIYTKCAYAC nosZ-II_R GCIGARCARAAITCBGTRC	697 bp	(95°C 5 min)x1 (95°C 15s, 54°C 30s, 72°C 30s, 80°C 10s)x40	Jones, Graf [12]

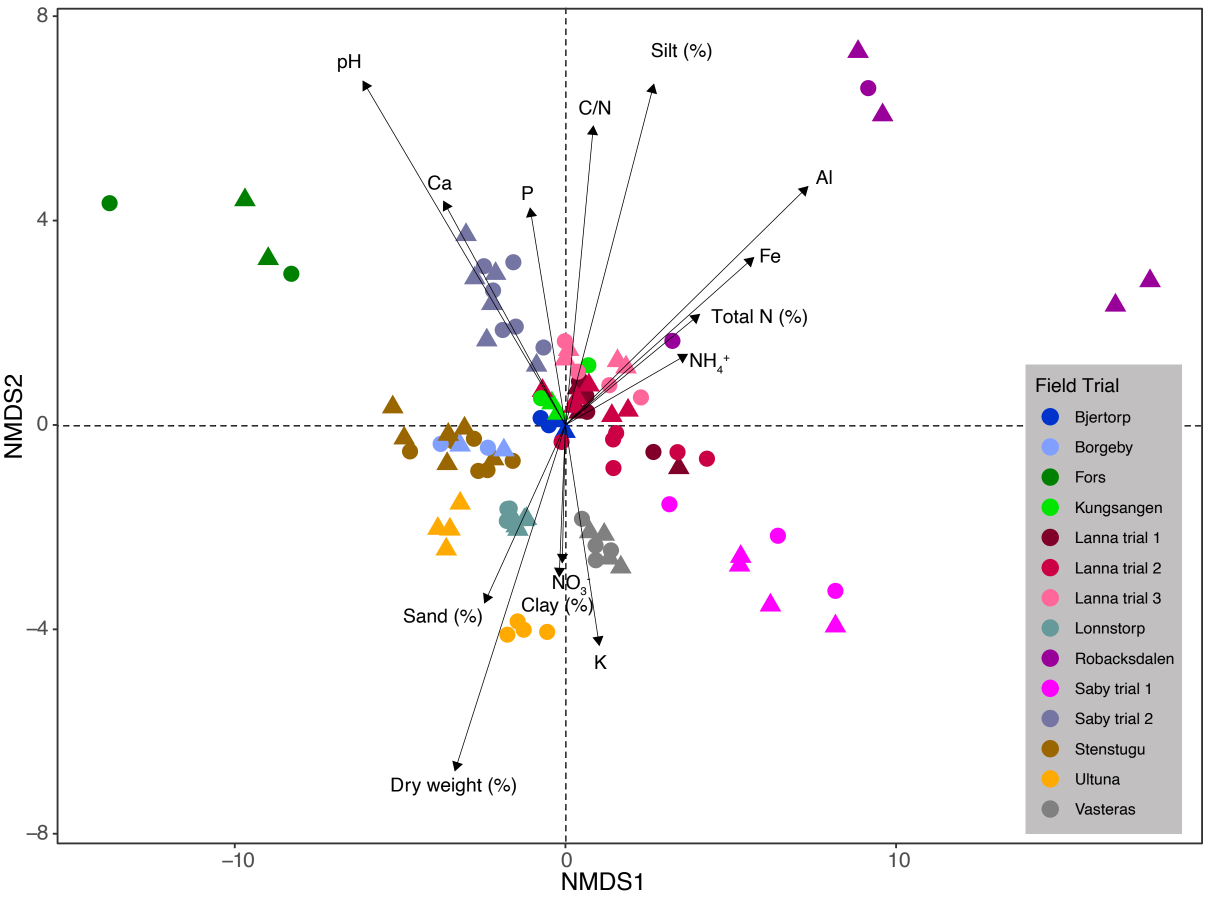

Supplementary Figure 1. Structure of microbial communities across 14 long-term N-fertilization field trials from different locations in Sweden. Ordination is based on non-metric multidimensional scaling (NMDS) of Euclidian distances generated from Phylogenetic isometric log-ratio (PhILR) transformed abundances of OTUs identified as being frequent members of the community, based on **Fig. S3**. Circles and triangles represent unfertilized and fertilized plots, respectively, from each field trial. Vector fitting was used to identify edaphic factors that were significantly ($p < 0.05$) correlated with differences in community structure across samples. Final stress of the NMDS ordination was 0.06.

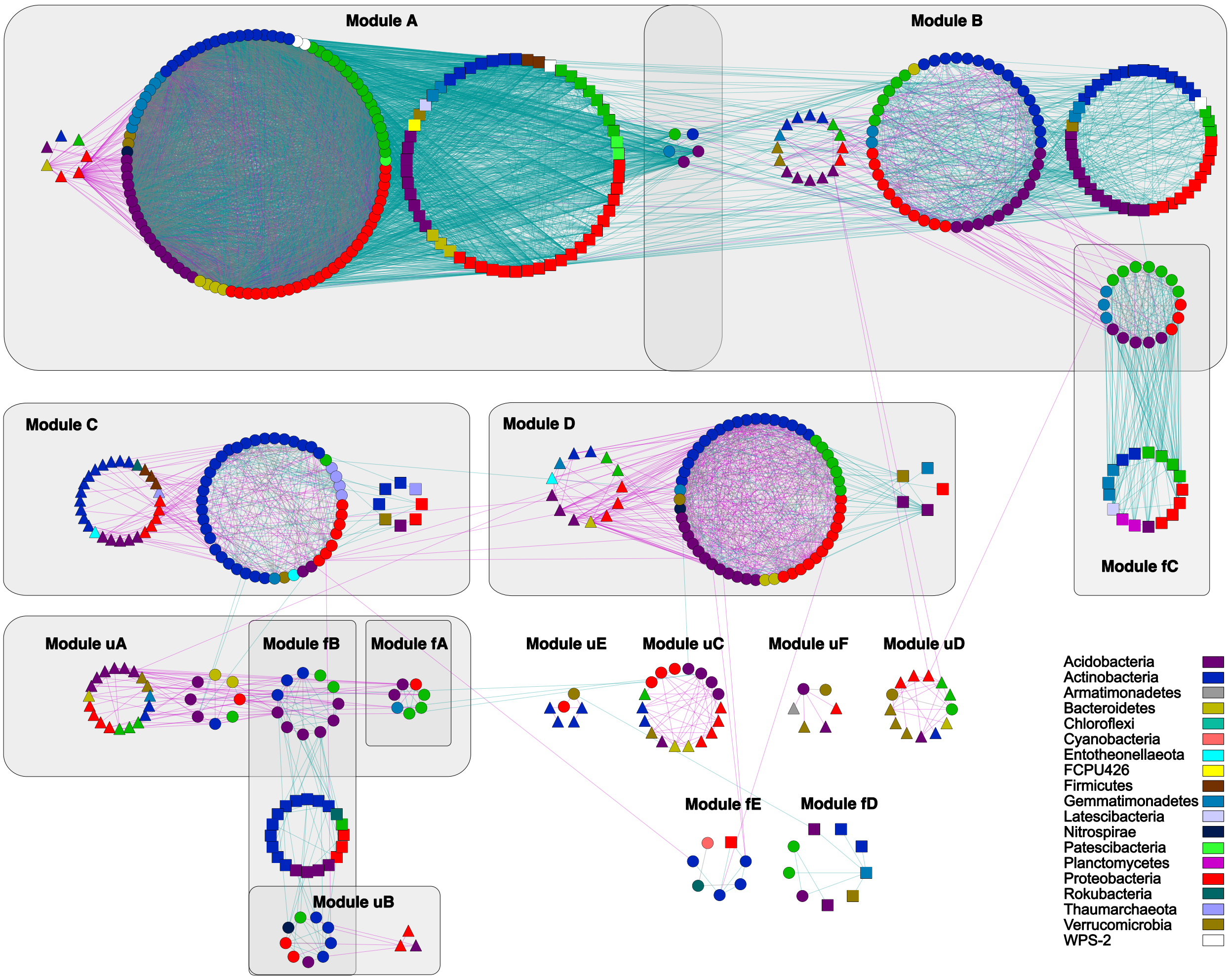

Module	Common (%)		Unfertilized (%)		Fertilized (%)	
	Nodes	Edges	Nodes	Edges	Nodes	Edges
A	61.7	29.2	4.2	10.5	34.1	60.3
B	53.8	16.0	12.1	20.7	34.1	63.3
C	55.6	26.6	34.6	40.3	9.9	33.1
D	75.0	22.9	18.1	59.6	6.9	17.5
uA	57.8	15.4	42.2	84.6	na	na
uB	75.0	53.8	25.0	46.2	na	na
uC	38.9	0.0	61.1	100.0	na	na
uD	15.4	4.5	84.6	95.5	na	na
uE	33.3	0.0	66.7	100.0	na	na
uF	33.3	20.0	66.7	80.0	na	na
fA	100.0	55.6	na	na	0.0	44.4
fB	50.0	20.0	na	na	50.0	80.0
fC	51.4	23.2	na	na	48.6	76.8
fD	33.3	12.5	na	na	66.7	87.5
fE	85.7	25.0	na	na	14.3	75.0

Supplementary Figure 2. Comparison of fertilized and unfertilized network topologies using Dynet. Nodes are grouped into modules detected in both fertilized and unfertilized networks, where node shape denotes whether nodes are found only in unfertilized (triangle) or fertilized (squares), or were common to both networks (circle). Edge color shows whether edges are unique to fertilized (purple) or unfertilized (turquoise) networks, or common to both (grey). Taxonomic affiliation of OTUs is indicated by node color. Nodes not included in modules are excluded for clarity. Percentage of nodes and edges that are common to each network within each module are shown in the table below.

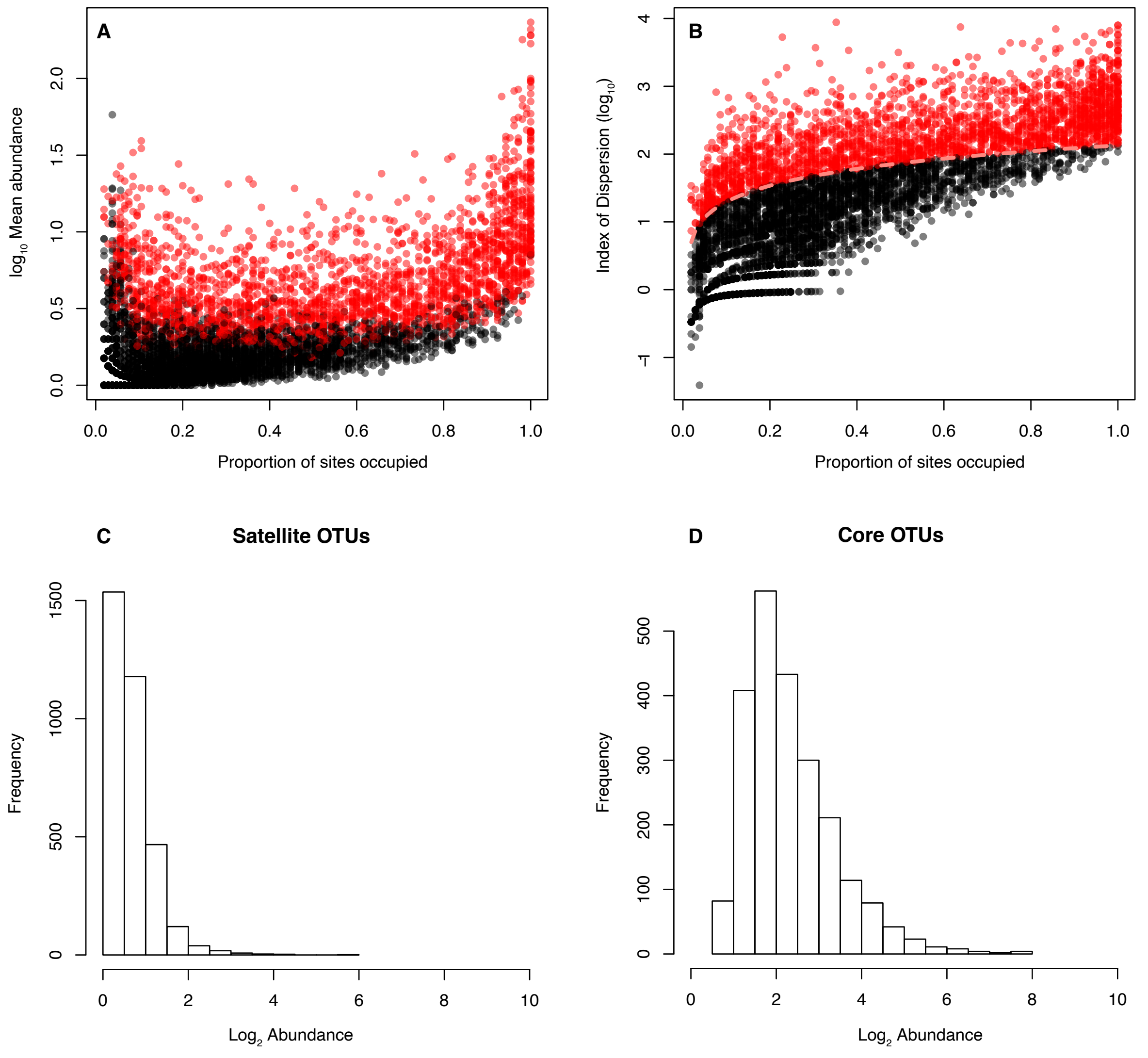

Supplementary Figure 3. Partitioning of total prokaryotic communities into frequent and rare OTUs based on species abundance distributions. A) Log mean abundance and B) index of dispersion of OTUs as a function of the proportion of sites occupied (red= frequent OTUs, black = rare OTUs). Core-satellite species theory predicts that rare OTU abundances are randomly (Poisson) distributed across sites and follow a log-series distribution (C), whereas frequently occurring OTU abundances are non-randomly distributed, resulting in index of dispersion values that significantly deviates from a χ^2 distribution and follow a log-normal distribution (D). The dashed pink line in B) denotes the 2.5 % confidence limit of the χ^2 distribution for the index of dispersion depending on occupancy.

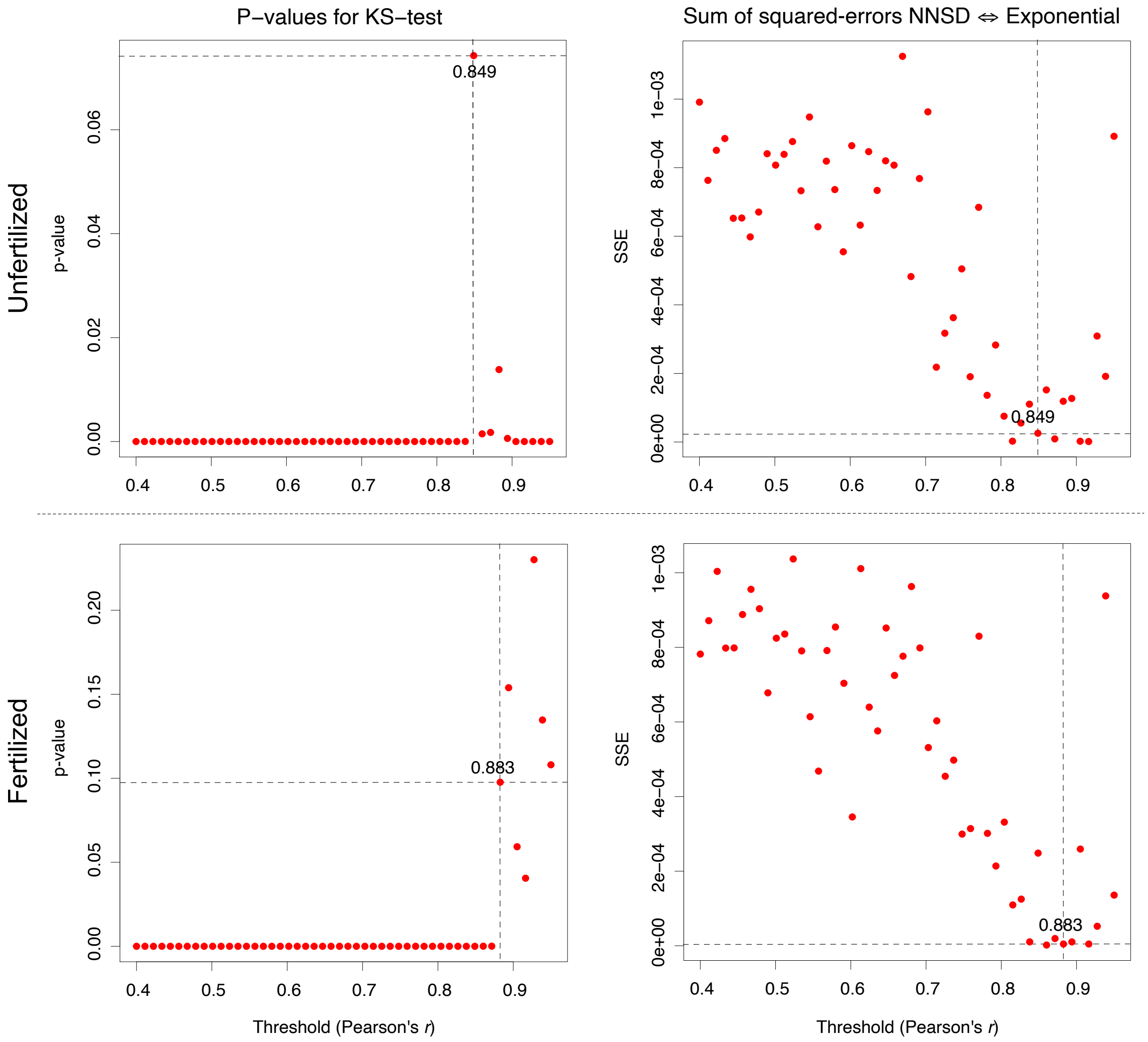

Supplementary Figure 4. Results of *RMThreshold*, which uses random matrix theory to define threshold values of Pearson's *r* for unfertilized and fertilized networks. Plots show Kolmorgorov-Smirnov (KS) tests (left) and sums of squared errors (SSE; right) comparing the empirical nearest neighbour spacing distributions (NNSD) and the theoretical exponential distribution over a range of threshold values of *r* for each dataset. Non-significant ($P>0.05$) KS-tests with minimal SSE indicate threshold values of *r* that result in exponential NNSDs, and therefore networks from which random noise has been removed.

Supplementary References

1. Kirchmann H, Persson J, Carlgren K. The Ultuna long-term soil organic matter experiment, 1956-1991. *Reports and dissertation* **17**, 1-55 (1994).

2. Carlgren K, Mattsson L. Swedish Soil Fertility Experiments. *Acta Agr Scand B-S P* **51**, 49-76 (2001).

3. Poeplau C, Kätterer T, Bolinder MA, Börjesson G, Berti A, Lugato E. Low stabilization of aboveground crop residue carbon in sandy soils of Swedish long-term experiments. *Geoderma* **237-238**, 246-255 (2015).

4. Persson T, Bergkvist G, Kätterer T. Long-term effects of crop rotations with and without perennial leys on soil carbon stocks and grain yields of winter wheat. *Nutr Cyc Agroecosys* **81**, 193-202 (2007).

5. Kätterer T, Börjesson G, Kirchmann H. Changes in organic carbon in topsoil and subsoil and microbial community composition caused by repeated additions of organic amendments and N fertilisation in a long-term field experiment in Sweden. *Agr Ecosyst Environ* **189**, 110-118 (2014).

6. Williams A, Börjesson G, Hedlund K. The effects of 55 years of different inorganic fertiliser regimes on soil properties and microbial community composition. *Soil Biol Biochem* **67**, 41-46 (2013).

7. Svensson K, Odlare M, Pell M. The fertilizing effect of compost and biogas residues from source separated household waste. *J Agri Sci* **142**, 461-467 (2004).

8. Lopez-Gutierrez JC, Henry S, Hallet S, Martin-Laurent F, Catroux G, Philippot L. Quantification of a novel group of nitrate-reducing bacteria in the environment by real-time PCR. *J Microbiol Meth* **57**, 399-407 (2004).

9. Throback IN, Enwall K, Jarvis A, Hallin S. Reassessing PCR primers targeting nirS, nirK and nosZ genes for community surveys of denitrifying bacteria with DGGE. *FEMS Microbiol Ecol* **49**, 401-417 (2004).

10. Henry S, Baudoin E, Lopez-Gutierrez JC, Martin-Laurent F, Brauman A, Philippot L. Quantification of denitrifying bacteria in soils by nirK gene targeted real-time PCR. *J Microbiol Meth* **59**, 327-335 (2004).

11. Henry S, Bru D, Stres B, Hallet S, Philippot L. Quantitative detection of the nosZ gene, encoding nitrous oxide reductase, and comparison of the abundances of 16S rRNA, narG, nirK, and nosZ genes in soils. *Appl Environ Microbiol* **72**, 5181-5189 (2006).

12. Jones CM, Graf DRH, Bru D, Philippot L, Hallin S. The unaccounted yet abundant nitrous oxide-reducing microbial community: a potential nitrous oxide sink. *ISME Jl* **7**, 417-426 (2013).
