# Supplementary material for: Reactive nitrogen restructures and weakens microbial controls of soil N2O emissions
Source: Commun Biol. 2022 Mar 28;5:273. doi: 10.1038/s42003-022-03211-4 (PMC8960841; doi:10.1038/s42003-022-03211-4)
Supplement: Supplementary file 3 — Description of Additional Supplementary Files [file 42003_2022_3211_MOESM3_ESM.pdf]

Description of Additional Supplementary Files

File name: Supplementary Data 1
Description: Supplemental table 7, Edaphic factors, denitrification and N_2O production rates and gene abundances

File name: Supplementary Data 2
Description: Supplemental table 8, OTU table

File name: Supplementary Data 3
Description: Supplemental table 9, Taxonomic identification
