# Supplementary material for: Reactive nitrogen restructures and weakens microbial controls of soil N2O emissions
Source: Commun Biol. 2022 Mar 28;5:273. doi: 10.1038/s42003-022-03211-4 (PMC8960841; doi:10.1038/s42003-022-03211-4)
Supplement: Supplementary file 5 — Reporting Summary [file 42003_2022_3211_MOESM5_ESM.pdf]

nature portfolio

Corresponding author(s): Sara Hallin

Last updated by author(s): Dec 16, 2021

Reporting Summary

Nature Portfolio wishes to improve the reproducibility of the work that we publish. This form provides structure for consistency and transparency in reporting. For further information on Nature Portfolio policies, see our Editorial Policies and the Editorial Policy Checklist.

Statistics

For all statistical analyses, confirm that the following items are present in the figure legend, table legend, main text, or Methods section.

n/a	Confirmed	
☐	☒	The exact sample size (n) for each experimental group/condition, given as a discrete number and unit of measurement
☐	☒	A statement on whether measurements were taken from distinct samples or whether the same sample was measured repeatedly
☐	☒	The statistical test(s) used AND whether they are one- or two-sided<br>*Only common tests should be described solely by name; describe more complex techniques in the Methods section.*
☐	☒	A description of all covariates tested
☐	☒	A description of any assumptions or corrections, such as tests of normality and adjustment for multiple comparisons
☐	☒	A full description of the statistical parameters including central tendency (e.g. means) or other basic estimates (e.g. regression coefficient) AND variation (e.g. standard deviation) or associated estimates of uncertainty (e.g. confidence intervals)
☐	☒	For null hypothesis testing, the test statistic (e.g. F, t, r) with confidence intervals, effect sizes, degrees of freedom and P value noted<br>*Give P values as exact values whenever suitable.*
☒	☐	For Bayesian analysis, information on the choice of priors and Markov chain Monte Carlo settings
☐	☒	For hierarchical and complex designs, identification of the appropriate level for tests and full reporting of outcomes
☐	☒	Estimates of effect sizes (e.g. Cohen's d, Pearson's r), indicating how they were calculated

Our web collection on statistics for biologists contains articles on many of the points above.

Software and code

Policy information about availability of computer code

Data collection	For quantitative PCR, CFX manager software was used to determine Ct curves obtained from CFX real-time PCR machines. Sequence data was obtain from SciLife labs using Illumina MiSeq software. Data from gas chromotography was collected using Perkin-Elmer Total chrome software. All software is proprietary commerical software.
Data analysis	For sequence data, read merging was performed using PEAR v0.9.8. Clustering of 16S rRNA sequence data into operational taxonomic units, including sequence quality filtering, chimera removal and clustering was performed using the VSEARCH v2.10.4 program. Generation of the phylogeny and taxonomy assignment of OTUs was performed using the ARB sequence analysis program in combination with the FastTree v2 program, in combination with the SILVA v132 database downloaded from the SILVA website. Visualization and network alignment was performed using Cytoscape v 3.5 and the Dynet Cytoscape plug-in application, respectively. All other analyses were performed using various published packages within the R v4.0 statistical software environment, and are referred to within the manuscript. Scripts for all analyses are available upon request.

For manuscripts utilizing custom algorithms or software that are central to the research but not yet described in published literature, software must be made available to editors and reviewers. We strongly encourage code deposition in a community repository (e.g. GitHub). See the Nature Portfolio guidelines for submitting code & software for further information.

Data

Policy information about availability of data

All manuscripts must include a data availability statement. This statement should provide the following information, where applicable:

- Accession codes, unique identifiers, or web links for publicly available datasets
- A description of any restrictions on data availability
- For clinical datasets or third party data, please ensure that the statement adheres to our policy

Sequence data is available from the Short Read Archive at the National Center for Biotechnology Information (NCBI) under BioProject accession PRJNA722868, and all relevant soil and gene abundance data are provided in a separate excel file as Supplemental Material Table S7.

Field-specific reporting

Please select the one below that is the best fit for your research. If you are not sure, read the appropriate sections before making your selection.

☐ Life sciences ☐ Behavioural & social sciences ☒ Ecological, evolutionary & environmental sciences

For a reference copy of the document with all sections, see nature.com/documents/nr-reporting-summary-flat.pdf

Ecological, evolutionary & environmental sciences study design

All studies must disclose on these points even when the disclosure is negative.

Study description	This study examines the link between changes in microbial community structure and potential N2O emissions from agricultural soils in response to long-term nitrogen fertilization. The study design is a survey of 14 different long-term fertilization trials established throughout Sweden, sampling from replicate plots that were either fertilized at typical fertilization rates (80-150 kg/ha) or unfertilized controls. As trials were established at different times by different researchers, the number of field replicates varied amongst the field trials, ranging from 2-6 replicates per trial (see supplemental table S1).
Research sample	The samples are composite soil samples obtained by combining five sub-samples of topsoil (0-20 cm) from each field replicate into a single soil sample, followed by sieving through a 4 mm sieve to homogenize the sample. Composite samples were used to provide the best representation of soil within each field replicate plot to obtain biological replicates for each field trial.
Sampling strategy	As the study seeks to identify general trends across multiple field sites, sample size was determined by the availability/accessibility of long-term field trials within Sweden that have been established to specifically look at the effects of fertilization.
Data collection	Quantitative PCR, sequencing, and potential activity data were collected by Tina Putz, Maren Tiemann and Christopher Jones using molecular and gas chromatography methods described in the materials and methods. All soil physico-chemical data was obtained through the use of a commercial, accredited soil analysis service (AgriLab AB, Uppsala Sweden).
Timing and spatial scale	Samples were obtained from 14 locations within Sweden, within an area ranging from 55° 40'N 13° 08'E to 63° 49'N 20° 13'E. Samples were taken at a single time point for each location within the period of November-December of 2013, after fields had been harvested. The dates of sampling for each site are as follows: Säby 1 & 2, 2013-11-01; Bjertorp, Lanna 1 2 & 3, 2013-11-07; Röbäcksdalen 2013-11-08; Borgeby & Stenstugu 2013-11-12; Lönnstorp 2013-11-22; Ultuna 2013-11-27; Fors, Kungsängen, Västerås 2013-12-04.
Data exclusions	We have excluded soil data for HCl extractable P and K and percent total C, as we determined these variables to be redundant and less relevant to Ammonium-Lactate extractable P and K, which better reflect the available concentrations of these resources to microorganisms in soil.
Reproducibility	The study is conducted as a field survey of a single time-point and is not conducive to experimental verification of reproducibility. However, all methods are based on well-established protocols, and full information on sampling locations and the methods used are provided in the manuscript.
Randomization	Randomization of samples was performed for all molecular methods by random assignment of sample order to DNA extracts from each soil sample. This was not necessary for potential activity or soil physico-chemical analyses.
Blinding	Not applicable.

Did the study involve field work? ☒ Yes ☐ No

Field work, collection and transport

Field conditions	Soil samples were obtained under ambiant field conditions during the months of october-november in 2014. Average temperature during this period ranged between 2-10°C depending on location. Cumulative precipitation ranged from 25-150 mm depending on location.

Location	Samples were obtained from multiple locations in Sweden. Coordinates for each sampling site are provided in table S1, supplemental materials.
Access & import/export	All long-term field sites were within Sweden and either managed by or working in collaboration with the Swedish University of Agricultural Sciences.
Disturbance	No disturbance was caused by the soil sampling methods used.

Reporting for specific materials, systems and methods

We require information from authors about some types of materials, experimental systems and methods used in many studies. Here, indicate whether each material, system or method listed is relevant to your study. If you are not sure if a list item applies to your research, read the appropriate section before selecting a response.

Materials & experimental systems

n/a	Involved in the study	
☒	☐	Antibodies
☒	☐	Eukaryotic cell lines
☒	☐	Palaeontology and archaeology
☒	☐	Animals and other organisms
☒	☐	Human research participants
☒	☐	Clinical data
☒	☐	Dual use research of concern

Methods

n/a	Involved in the study	
☒	☐	ChIP-seq
☒	☐	Flow cytometry
☒	☐	MRI-based neuroimaging
